# A protocol for developing, disseminating, and implementing a core outcome set (COS) for childbirth pelvic floor trauma research

**DOI:** 10.1186/s12884-020-03070-z

**Published:** 2020-06-26

**Authors:** Stergios K. Doumouchtsis, Maria Patricia Rada, Vasilios Pergialiotis, Gabriele Falconi, Jorge Milhem Haddad, Cornelia Betschart

**Affiliations:** 1 Department of Obstetrics and Gynaecology, Epsom & St Helier University Hospitals NHS Trust, London, UK; 2 Laboratory of Experimental Surgery and Surgical Research N S Christeas, Athens University Medical School, Athens, Greece; 3 St George’s University of London, London, UK; 4 American University of the Caribbean School of Medicine, Coral Gables, USA; 5 CHORUS, an International Collaboration for Harmonising Outcomes, Research and Standards in Urogynaecology and Women’s Health, London, UK; 6 2nd Department of Obstetrics-Gynaecology, “Dominic Stanca” Clinic, “Iuliu Hatieganu” University of Medicine and Pharmacy Cluj-Napoca, Cluj-Napoca, Romania; 7 Department of Obstetrics and Gynaecology, San Bortolo Hospital, Vicenza, Italy; 8 Department Obstetrics and Gynaecology, Urogynaecology Division, Hospital das Clínicas da Faculdade de Medicina da Universidade de São Paulo, São Paulo, Brazil; 9 Department of Gynecology, University Hospital of Zurich, Frauenklinikstrasse 10, CH-8091 Zurich, Switzerland

**Keywords:** Childbirth pelvic floor trauma, Birth-related injuries, Obstetric anal sphincter injury (OASIS), Urinary incontinence (UI), Levator Ani muscle (LAM), International collaboration for Harmonising outcomes (CHORUS), Core outcome measures in effectiveness trials (COMET), Core outcome set (COS), Recommendations for interventional trials (SPIRIT)

## Abstract

**Background:**

More than 85% of women sustain different degrees of trauma during vaginal birth. Randomized controlled trials on childbirth pelvic floor trauma have reported a wide range of outcomes and used different outcome measures. This variation restricts effective data synthesis, impairing the ability of research to inform clinical practice. The development and use of a core outcome set (COS) for childbirth pelvic floor trauma aims to ensure consistent use of outcome measures and reporting of outcomes.

**Methods:**

An international steering group, within CHORUS, an International Collaboration for Harmonising Outcomes, Research and Standards in Urogynaecology and Women’s Health, including academic community members, researchers, healthcare professionals, policy makers and women with childbirth pelvic floor trauma will lead the development of this COS. Relevant outcome parameters will be identified through comprehensive literature reviews. The selected outcomes will be entered into an international, multi-perspective online Delphi survey. Subsequently and based on the results of the Delphi surveys consensus will be sought on ‘core’ outcomes.

**Discussion:**

Dissemination and implementation of the resulting COS within an international context will be supported and promoted. Embedding the COS for childbirth pelvic floor trauma within future clinical trials, systematic reviews, and clinical practice guidelines is expected to enrich opportunities for comparison of future clinical trials and allow better synthesis of outcomes, and will enhance mother and child care. The infrastructure created by developing a COS for childbirth pelvic floor trauma could be leveraged in other settings, for example, advancing research priorities and clinical practice guideline development.

## Background

Childbirth pelvic floor trauma affects more than 85% of women during vaginal birth [1]. The incidence of different degrees of perineal trauma vary from over 70% in multiparous to over 91% in nulliparous women [1]. The physical consequences of vaginal childbirth may be mild, subclinical conditions or severe pathologies either immediately or in the long term.

Childbirth pelvic floor trauma commonly refers to perineal and vaginal trauma following delivery and the focus of research has been on the perineal body and the anal sphincter complex. However, it may affect different anatomical structures of the pelvic floor including muscles, nerves, connective tissue, as well as bone trauma. Such trauma can involve rupture, compression and stretching of different tissues and organs of the pelvis and pelvic floor resulting in nerve, muscle and connective tissue damage.

The incidence of urinary incontinence (UI) six months postpartum was 20.7% in a recent large cohort study [2]. A clinical diagnosis of obstetric anal sphincter injury (OASIS) is made in between 1 and 11% of women having a vaginal delivery [3, 4]. The reported incidence of levator ani muscle (LAM) trauma varies widely and has been reported to range between 13 and 26% in women who have a vaginal delivery [5–8]. Short and long-term morbidity associated with childbirth pelvic floor trauma can have a significant effect on daily activities, psychological wellbeing, sexual function and overall quality of life. The development of therapeutic interventions to reduce this health burden is therefore urgently required to generate further evidence in order to inform clinical practice.

Childbirth pelvic floor trauma and associated outcomes have been defined using different clinical symptoms, diagnostic parameters and outcome measures and there is no international consensus regarding the assessment and management of different types of childbirth pelvic floor trauma. In the absence of a standardized approach, significant outcomes may not be properly collected and reported. Evidence synthesis can be further limited by the use of different outcome measures (including definitions and instruments).

For example, a Cochrane Review suggested that different pushing strategies showed no difference in perineal laceration and episiotomy, neither in resulting in caesarean and instrumental deliveries, nor in the neonatal outcomes. Interestingly, delayed pushing in labor led to a shortening of the actual time pushing and increased rates of spontaneous vaginal delivery whereas an overall longer second stage may be a risk factor for pelvic floor trauma. However, the studies included were admittedly of moderate to low quality and failed to show any clear difference in serious perineal trauma and episiotomy rates [9]. Another Cochrane review concluded that the effectiveness of interventions for women in subsequent pregnancies following obstetric anal sphincter injury is unknown [10]. Possible pre- and intrapartum interventions to consider are pelvic floor muscle training, perineal massage, balloon dilatation, warm compresses, birthing positions and pushing strategies that may impact pelvic floor trauma [11, 12]. The main issues that have been highlighted in previous systematic reviews including the one our group published recently [13] are inconsistent selection, measurement and reporting of outcomes.

Addressing the variation in outcome selection and reporting should be a priority, therefore the aim of this initiative is to develop, disseminate, and implement a core outcome set (COS) for childbirth pelvic floor trauma research. Specific projects led by CHORUS, an International Collaboration for Harmonising Outcomes, Research and Standards in Urogynaecology and Women’s Health aim to tackle such limitations in research evidence. A systematic review evaluating the variations in outcome measures and outcome reporting in childbirth pelvic floor trauma trials has been completed and published [13].

This project has been prospectively registered with the Core Outcome Measures in Effectiveness Trials [COMET] initiative, registration number 981 [14]. The Core Outcomes in Women’s Health [CROWN] initiative [www.crown-initiative.org] will support the dissemination and implementation of a COS for childbirth pelvic floor trauma to increase the value of primary research by encouraging future childbirth pelvic floor trauma trials to report core outcomes and, therefore, contribute data to high quality meta-analyses [15].

The aim of the study is the development and future implementation of a core outcome set (COS) for childbirth pelvic floor trauma research. A multiperspective group of professionals, patients and researchers will contribute to the development of a COS following a standard process. Such a COS will strengthen the consistent reporting of outcomes and use of outcome measures.

## Methods

### Scope of the COS for childbirth pelvic floor trauma

This COS will apply to clinical studies evaluating interventions for the management of women with childbirth pelvic floor trauma. All therapeutic interventions for childbirth pelvic floor trauma will be considered regardless of type, setting, or mode of administration. In order to cover the entire spectrum of degrees of perineal trauma, we will not differentiate between specific types of trauma. Childbirth pelvic floor trauma will be defined as any trauma to the pelvic floor, perineum, anal sphincter complex, lower urogenital tract and lower gastrointestinal tract sustained at the time of vaginal childbirth. The scope of the COS goes beyond interventions for the management of pelvic floor trauma to the direction of prevention, prevention studies and mixed studies, too.

### Study design

The development of this protocol was guided by the COMET Handbook [16] and was undertaken with consideration of the COS-STAndards for Reporting (COS-STAR) statement and checklist [17], albeit specific adaptations have been made to meet the aims of this project.

**Fig. 1:**
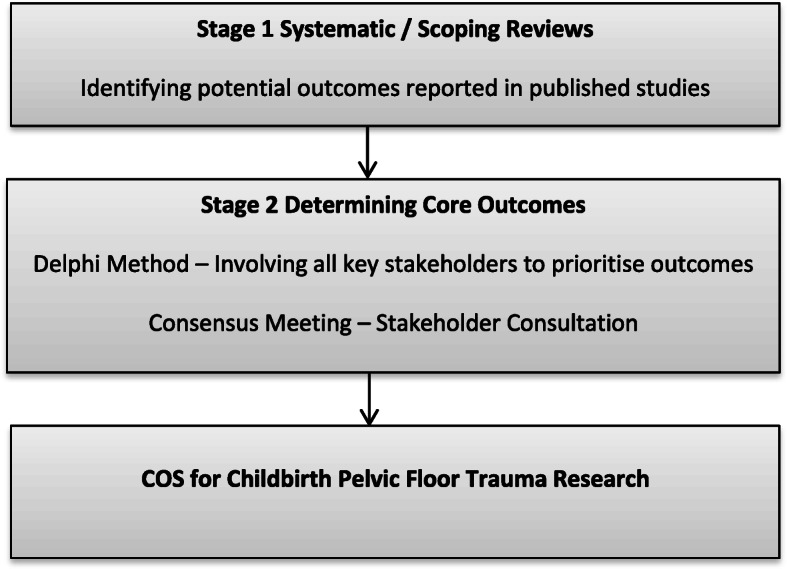
Development steps of COS for childbirth pelvic floor trauma research

A steering committee co-ordinated by CHORUS will guide all steps involved in the development of this COS for childbirth pelvic floor trauma (Fig. 1).

### Stage 1: systematic / scoping review (identifying potential outcomes)

Creating a comprehensive inventory of outcomes and outcome measures enables the identification of key concepts and the evaluation of the extent of existing variations in outcome reporting in childbirth pelvic floor trauma research. Based on our previous work [13, 14] the outcomes listed in Table 1 will be included into a modified Delphi survey. Any stakeholder can bring in terms on interventions for the prevention that might not be covered by the publications [13, 14]. Within the Delphi process and subsequent multi-perspective consensus meetings, any gaps in previously identified and evaluated potentially eligible outcomes will be reviewed and considered.

**Table 1:** Inventory of outcomes. PRO patient-reported outcome, EMG electromyography

Outcome	Diagnostic criteria
Anal USS abnormality	Imaging parameters
Anal manometry abnormality	Pressure measures
Anal incontinence	Scores, questionnaires, clinical examination, incl. Palpation, manometry
Defecatory difficulties	Scores, questionnaires, clinical examination, imaging, manometry
Flatus incontinence	Scores, questionnaires, clinical examination, manometry
Faecal urgency	Scores, questionnaires, clinical examination, imaging, manometry
Dyspareunia	Scores, questionnaires, clinical examination (cotton swab test, von Frey Filaments test)
Time of resumed intercourse	Personal history
Perineal pain	Personal history, clinical examination, imaging
Need for analgesia postnatally	Data from clinic information systems, personal history
Need to remove sutures	Data from clinic information systems, personal history
Need for resuturing	Data from clinic information systems, personal history
Wound dehiscence	Imaging, pictures, clinical examination
Wound healing	Imaging, pictures, clinical examination
Wound infection	Imaging, pictures, clinical examination
Wound gapping	Imaging, pictures, clinical examination
Levator ani muscle injury	Palpation, imaging, EMG
Women’s postpartum mobility	PRO, questionnaires
Women’s postpartum self-care	PRO, questionnaires
Activities of daily living	PRO, questionnaires
Caring for the newborn	PRO, questionnaires

### Outcome selection for use in clinical trials of childbirth pelvic floor trauma

Selection of appropriate outcomes is an essential for study design. Clinical trials that evaluate benefits and harms of interventions for childbirth pelvic floor trauma must select outcomes of relevance to key stakeholders and measure them using appropriate tools. The main issues that arise throughout this process are inconsistent selection, measurement and reporting of outcomes. Measuring outcomes of interventions for childbirth pelvic floor trauma in a variety of ways leads to outcome reporting bias. Therefore, the barrier to compare and highlight differences in research findings has an inevitable and negative impact on their interpretation and embedding into clinical practice.

The Standard Protocol Items: Recommendations for Interventional Trials (SPIRIT) statement, supported by funders of health research, recommends the use of COS where they exist [18]. Mapping all outcomes reported in clinical trials on interventions for childbirth pelvic floor trauma will provide the basis for initializing the process of development of COS.

Outcomes will be listed in a database, categorized in domains and themes and coded according to the taxonomy proposed by the COMET Initiative [16]. If there is uncertainty as to how to classify or present an outcome, consensus of the steering group will be sought. Following the steering group’s agreement, the outcome inventory will be entered into the Delphi method.

### Stage 2. Determining core outcomes

#### Creating a group for the development of a COS for childbirth pelvic floor trauma

A multiperspective group of healthcare professionals, researchers and women who sustained childbirth pelvic floor trauma will contribute to the development of a COS that will be applicable to research evaluating outcomes and outcome measures for childbirth pelvic floor trauma.

This group will consider the urgent need for development of effective interventions to reduce the impact of childbirth pelvic floor trauma on women’s quality of life given the flaws and weaknesses of current evidence documented in Cochrane reviews [19] [20] [21].

### Research management

The project for developing a COS for childbirth pelvic floor trauma will be guided by a management team and a steering committee. The management team will include research partners who will meet regularly and organize daily tasks and overall work progress. The steering committee will meet at 6 monthly intervals. A coordinator and two other expert members will provide advice on methodology and childbirth pelvic floor trauma-related issues. There will also be representatives from the management team. The purpose of the steering committee will be to provide support and guidance on any arising issues. Two women with childbirth pelvic floor trauma will be invited to participate in the study management and oversight of the process.

### Delphi survey

A modified Delphi approach is a method where several surveys are delivered over a series of rounds [22]. This sequential, robust method has a significant advantage over other less structured consensus methods. Web-based Delphi surveys facilitate stakeholders participation being feasible and efficient [23, 24]. Potential key stakeholders as listed in Table 2 will be identified through appropriate contact methods and invited to participate in the Delphi survey. Representation will be aimed for each stakeholder group.

**Table 2:** Key stakeholders

Stakeholders	Definition
Women with experience of childbirth pelvic floor trauma	Parous women with history of pelvic floor trauma
Clinicians	Physiotherapists, obstetricians, gynaecologists and urogynaecologists with clinical experience in their field actively practising at present
Researchers	Pelvic floor research, focus on peripartum pelvic floor research
Pharmaceutical industry representatives	Representatives from pharmaceutical industry actively involved in the area of childbirth pelvic floor trauma
Professional organizations representatives	Representatives from professional organisations with relevant scope and practice
Policy makers	Representatives from organisations that implement policies in this field
Healthcare regulators	Representatives from regulatory bodies

A snowball sampling approach will be used to identify potential participants in different stakeholder groups who will be invited via electronic communication tools (email or social media). Researchers and healthcare professionals will be identified through relevant published papers and national and international professional organizations. Policy makers will be identified through published reports and policies. Public representatives will be identified and invited to participate through patient groups and social media. Relevant organizations will be approached and encouraged to distribute the invitation to their members. The research team will provide these organizations with information about the aims of the study, the importance of developing core outcome sets and the methodology and process.

The invitation email will also contain an electronic link to the CHORUS portal, which will allow stakeholders to register for the online Delphi survey. We aim to recruit similar numbers of participants to each stakeholder group. There are no clear recommendations for calculating the required sample [25]. However, based upon previous studies, we aim to include 20 participants from each stakeholder group.

The number of responder participants in each stakeholder group will be reviewed following the end of round 1. Results will be presented as total number and / or percentage of:

- registrations
- respondents who have completed the survey
- respondents who completed the round
- respondents in each stakeholder group
- respondents compared to potential respondents as identified from the information provided by the steering committee
- new respondents who were not included in the original invitation to complete the survey

### Round 1

Participants will be asked to register online, provide demographic details, and commit to all rounds [26]. They will be allocated a unique identifier, which will make their responses anonymous.

Participants will be asked to score individual outcomes using a 7-point Likert Scale anchored between 1 (not important) to 7 (critical). This scale was created by the Grading of Recommendations Assessment, Development and Evaluation (GRADE) working group and has been widely adopted by COS Developers [27]. During the first round, participants will be invited to suggest additional outcomes. The round will close following a 4-week window. Additional outcomes listed by participants will be reviewed by the outcome committee and, if novel, listed in round two.

### Round 2

Participants will present their individual and stakeholder group responses and they will be invited to reflect on the observed results before proceeding to the next step and scoring individual outcomes again. The round will close following a 4-week window. The modified Delphi method promotes repeated reflection, rescoring of outcomes and facilitates stakeholder group agreement with regards to “core” outcomes [28]. This round’s results will enable individual outcomes to be classified as shown in Table 3. Although subjective, these definitions and criteria have been proposed by previous COS developers [25] (21) [29] and help with collecting uniform data.

**Table 3:** Consensus status based on core outcome criteria

Consensus status	Description	Criteria
Consensus in	Classify as a core outcome	Over 70% of participants in each stakeholder group score this outcome domain ‘critical’ AND Less than 15% of participants in each stakeholder group score outcome domain ‘not important’
Consensus out	Do not classify as a core outcome	Over 70% of participants in each stakeholder group score outcome domain ‘not important’ AND Less than 15% of participants in each stakeholder group score outcome domain ‘critical’
Lack of consensus	Do not classify as a core outcome	Anything else

The round two results will be reviewed by the steering group to consider the need for a further Delphi survey round.

### Stakeholder consultation

During this final phase, a meeting conducted by an independent coordinator will be organized with the purpose of deciding which outcomes will be validated and included in the COS. In addition, outcomes that do not meet core outcome criteria will be discussed. This meeting will purposefully include various points of view from participants who have completed all rounds of the Delphi survey. During the consensus meeting, the results from each round of the Delphi survey will be presented. To avoid biased consensus formation amongst a group of varied participants, the steering committee will consider all opinions [15, 29] in an interactive meeting. To facilitate dissemination and implementation, editors from key journals and funders of childbirth pelvic floor trauma research will be invited to participate.

### Statistics

The Delphi consensus is defined in a priori specification in Table 3. Each core outcome will be classified within a threshold value of > 70% of participants considering the core outcome parameter as critical, or not important, or lack of consensus what results in a non-classification as a core outcome parameter.

The analyses will be primarily descriptive, with frequency counts provided for the variables. A limited number of analyses for trends within categorical variables (chi-square or Fisher’s exact test) will be performed. These analyses examine the relationship between measures of consensus, the different stakeholders and diagnostic criteria.

### Ethical and governance considerations

As with previous COS development projects, this project is considered as a service evaluation not influencing patient safety [30–32]. Moreover, the Medical Research Council decision tool [33] indicated that this study protocol does not require NHS Research Ethics Committee (REC) approval.

All participants involved will be asked for their consent before participating in either stakeholder interviews or the Delphi survey, and all procedures will be conducted according to the Declaration of Helsinki [32]. A “no-response” option will be allowed both for the survey and interactive parts of the research to ensure responder’s right to withhold information. A specific timeframe of the Delphi process will be provided and information concerning the interval of data storage and handling will be made available to participants.

## Discussion

This protocol for the development of COS for childbirth pelvic floor trauma adheres to best practice guidance provided by the COMET Ηandbook being in line with CHORUS’s aims and with other protocols which have adopted COS methodologies for various health conditions.

The development of COS incorporates the perspectives of multiple stakeholders including academic community members, researchers, health care professionals, policy makers and patient communities. This approach will ensure that the views, priorities and interests of all groups are considered in the selection of outcomes and outcome measures in childbirth pelvic floor trauma trials in the future.

Due to the miscellany of terms and definitions, it will be of importance to capture a wide range of terms also apart RCT. Any term or definition brought up will be assessed in a multiperspective view by the different stakeholders. As many core areas of the impact of childbirth trauma on women’s well-being may have not been addressed in currently available evidence, the whole process will encourage the identification and consideration of any wider physical and psychosocial sequelae which in turn are interrelated and may influence each other in order to ensure key outcomes are include in a COS.

Based on this process, a better methodology in selecting collecting and reporting outcomes in childbirth pelvic floor trauma research could enhance the quality of published research and reduce bias and research waste.

The National Institute for Health and Care Excellence supports the use of COS when selecting outcomes during evidence scoping and synthesis [34]. As this activity forms the basis of guideline development, the COS could have a direct impact in improving healthcare of women with childbirth pelvic floor trauma.

### Strengths and limitations

This protocol represents the first international, multiprofessional and multidisciplinary initiative to develop a core outcome set (COS) for use in childbirth pelvic floor trauma clinical trials. With a documented wide variation in the reported outcomes and outcome measures impairing our ability to synthesize primary research, as well as various methodological flaws in currently available research evidence, the development of this COS is highly warranted. The process to develop the COS is well established and involves a rigorous methodology, with extensive literature reviews, wide stakeholder participation, Delphi survey and a consensus meeting to establish the COS. This approach will encourage inclusion of diverse perspectives internationally.

However, the selected COS may not be applicable to all types of research or methodologies and the development of additional COS or updated ones may be warranted following this process.

### Project status

Part of stage 1 (systematic review on the variations of reported outcomes) has been completed and published [13]. The investigators have had online consultations on the development and drafting of this protocol. The first round of the e-Delphi survey is currently being developed but has not yet been completed. Protocol modifications may be implemented following consensus among the investigators if logistic issues arise.

## Data Availability

Not applicable as it is a protocol article. Future data will be stored in an open-repository, university based.

## Abbreviations

CHORUS: International Collaboration for Harmonising Outcomes
COMET: Core Outcome Measures in Effectiveness Trials
COS: Core Outcome Set
COS-STAR: COS-STAndards for Reporting
LAM: Levator Ani Muscle
OASIS: Obstetric Anal Sphincter Injury
REC: Research Ethics Committee
UI: Urinary Incontinence
